# Causal effects of education, intelligence, and income on COVID-19: evidence from a Mendelian randomization study

**DOI:** 10.1186/s40246-025-00731-y

**Published:** 2025-02-25

**Authors:** Yuqing Song, Ancha Baranova, Hongbao Cao, Weihua Yue, Fuquan Zhang

**Affiliations:** 1 Institute of Mental Health, Peking University Sixth Hospital, Beijing, 100191 China; 2 NHC Key Laboratory of Mental Health (Peking University), National Clinical Research Center for Mental Disorders (Peking University Sixth Hospital), Beijing, 100191 China; 3 School of Systems Biology, George Mason University, Manassas, 20110 USA; 4 Research Centre for Medical Genetics, Moscow, 115478 Russia; 5 McGovern Institute for Brain Research, PKU-IDG, Peking University, Beijing, 100871 China; 6 Chinese Institute for Brain Research, Beijing, 102206 China; 7 Institute of Neuropsychiatry, The Affiliated Brain Hospital of Nanjing Medical University, Nanjing, 210029 China; 8 Department of Psychiatry, The Affiliated Brain Hospital of Nanjing Medical University, Nanjing, 210029 China

**Keywords:** COVID-19, Educational attainment, Intelligence, Income, Socioeconomic status, Mendelian randomization, Genetics

## Abstract

**Background:**

The protective effects of higher educational attainment (EA) and intelligence on COVID-19 outcomes are not yet understood with regard to their dependency on income. The objective of our study was to examine the overall as well as independent effects of the three psychosocial factors on the susceptibility to and severity of COVID-19. To accomplish this, we utilized genetic correlation, Mendelian randomization (MR), and multivariable MR (MVMR) analyses to evaluate genetic associations between EA, intelligence, household income, and three specific COVID-19 outcomes: SARS-CoV-2 infection, hospitalized COVID-19, and critical COVID-19.

**Results:**

The genetic correlation analysis revealed that COVID-19 outcomes were negatively correlated with the three psychosocial factors (r_g_: -0.19‒-0.36). The MR analysis indicated that genetic liability to EA, intelligence, and income exerted overall protective effects against SARS-CoV-2 infection (OR: 0.86‒0.92), hospitalized COVID-19 (OR: 0.70‒0.80), and critical COVID-19 (OR: 0.65‒0.85). MVMR analysis revealed that elevated levels of EA conferred independent protective effects against SARS-CoV-2 infection (OR: 0.85), hospitalization due to COVID-19 (OR: 0.79), and critical COVID-19 (OR: 0.63). Furthermore, intelligence exhibited a negative association with the risk of SARS-CoV-2 infection (OR: 0.91), whereas a higher income was linked to an elevated risk of SARS-CoV-2 infection (OR: 1.13).

**Conclusions:**

Our findings indicated that EA could significantly reduce the risk and severity of COVID-19, regardless of intelligence and income. However, the impact of intelligence or income on COVID-19 severity was not supported by our research.

**Supplementary Information:**

The online version contains supplementary material available at 10.1186/s40246-025-00731-y.

## Background

Since December 2019, coronavirus disease 2019 (COVID-19) has spread all over the world, seriously affecting human health and communication. Risk and protective factors have been reported to be associated with the susceptibility or severity of COVID-19 [1–5]. Meanwhile, COVID-19 can lead to a myriad of post-COVID-19 consequences [6–11]. An individual’s ability to maintain health is not limited to the personal physical quality or access to medical care but depends on many psychosocial factors, policy formulation, and personal cognitive levels [12]. From prevalence to mortality, the epidemiology of COVID-19 is also affected by the socioeconomic or psychological status of exposed individuals [13, 14]. Indeed, the infection and severity rates of COVID-19 in the population of low socioeconomic status were much higher than in those with higher socioeconomic levels [15].

Educational attainment (EA), intelligence, and income are factors associated with socioeconomic and psychological status [12, 16]. It was reported that the mortality from infectious diseases in individuals with lower education levels was approximately twofold higher than that of individuals with higher educational levels [17]. A population-based retrospective cohort study in California found a higher mortality rate of COVID-19 in individuals with lower EA [18]. A cross-sectional study in India found that COVID-19-infected patients with a college degree or higher had less severe in-hospital outcomes and mortality than those with no college-level education [19]. In Brazil, poor patients were more likely to be hospitalized due to COVID-19, while individuals of African descent and/or low EA individuals were more likely to have comorbidities [20]. These findings may be explained by differential access to COVID-19 information, by variation in prevention strategies, and by greater availability of on-demand health care in socioeconomically advanced population groups.

A nationwide cohort study in South Korea found that a high-income level was reflected by lower odds of COVID-19 infection but not by differences in COVID-19 morbidity and mortality [21]. In Brazil’s first wave of COVID-19 infection, there were higher infection rates in the poorest nonwhite populations residing in the Northern and Northeastern parts of the country [22]. A cross-sectional study in Iran found a significant and positive relationship between recent declines in income and COVID-19 hospitalization [23]. All previous studies described the association between low socioeconomic status and high risk of COVID-19 as being influenced by many external factors, such as the immediate environment and resource availability.

In a two-sample Mendelian randomization (MR) study, the link between a genetic predisposition to higher EA and intelligence and a reduced risk of contracting COVID-19 was reported recently [24, 25], while associations of EA with COVID-19 hospitalizations were not examined [24]. Another study found that EA could protect individuals from developing either hospitalized or critical COVID-19, while intelligence protected them from hospitalized COVID-19 only but not from SARS-CoV-2 infection [26]. All of the above studies examined only the causal effect of either EA or intelligence on the outcomes of COVID-19, with no consideration of the effects of income.

Here, we hypothesize that the overall effects of each of the three psychosocial factors on COVID-19 may be partially mediated or confounded by the other two factors, and the direct effects of each factor may differ from their overall effects. To test this hypothesis, we performed MR and multivariable MR (MVMR) analyses and compared the overall and independent effects of EA, intelligence, and income on COVID-19 outcomes. MR studies use genetic variants, typically single-nucleotide polymorphisms (SNPs), that are reliably associated with exposures of interest but do not vary along with confounders. MR is a robust method to test the causality between an exposure (such as EA) and an outcome (for example, SARS-CoV-2 infection) [27].

## Methods

### Study design and data sources

Our study was based on publicly available GWAS summary results. The summary statistics for the outcomes of COVID-19 were obtained from the COVID-19 Host Genetics Initiative (HGI) GWAS meta-analysis round 7, including SARS-CoV-2 infection (122,616 cases and 2,475,240 controls), hospitalized COVID-19 (32,519 cases and 2,062,805 controls), and critical COVID-19 (13,769 cases and 1,072,442 controls) [28]. The SARS-CoV-2 infection dataset mainly reflects the overall susceptibility to the virus, whereas the hospitalized and critical COVID-19 datasets represent the severity of the disease. The GWAS datasets for EA [29], intelligence [30], and household income [31] included 765,283, 269,867, and 392,422 participants, respectively. Information about the data sources and sample sizes is summarized in Table 1. The study was written according to the 20-item STROBE-MR checklist (Supplementary Table S1).

**Table 1:** Summary information of the datasets

No	Trait	Year	First Author	PMID	Ncase	Ncontrol	*N*
1	EA	2022	Okbay A	35,361,970	NA	NA	765,283
2	Intelligence	2018	Savage JE	29,942,086	NA	NA	269,867
3	Income	2019	Jiang L	31,768,069	NA	NA	392,422
4	SARS-CoV-2 infection	2021	COVID-19 HGI	32,404,885	122,616	2,475,240	2,597,856
5	Hospitalized COVID-19	2021	COVID-19 HGI	32,404,885	32,519	2,062,805	2,095,324
6	Critical COVID-19	2021	COVID-19 HGI	32,404,885	13,769	1,072,442	1,086,211

EA: Educational attainment

### Genetic correlation analysis

The genetic correlations between EA, intelligence, income, and the three outcomes of COVID-19 were calculated by LD score regression (Table 2). A set of SNPs was filtered down to 1.1 million variants, a subset of 1000 Genomes and HapMap3, with MAF above 0.05. Significant genetic correlations were determined after applying the correction for the false discovery rate (FDR < 0.05).

**Table 2:** Genetic correlations between education attainment, intelligence, and income and COVID-19 outcomes

Exposure	Outcome	*r*_g_ (se)	Z	*P*	FDR
EA	SARS-CoV-2 infection	-0.36 (0.04)	-9.15	5.49E-20	4.94E-19
Income	SARS-CoV-2 infection	-0.19 (0.05)	-4.06	4.92E-05	5.54E-05
Intelligence	SARS-CoV-2 infection	-0.31 (0.05)	-6.07	1.26E-09	3.78E-09
EA	Hospitalized COVID-19	-0.32 (0.05)	-6.94	3.89E-12	1.75E-11
Income	Hospitalized COVID-19	-0.24 (0.05)	-5.25	1.55E-07	2.79E-07
Intelligence	Hospitalized COVID-19	-0.25 (0.05)	-4.62	3.88E-06	5.82E-06
EA	Critical COVID-19	-0.28 (0.05)	-5.58	2.42E-08	5.45E-08
Income	Critical COVID-19	-0.26 (0.06)	-4.51	6.44E-06	8.28E-06
Intelligence	Critical COVID-19	-0.22 (0.06)	-3.61	3.12E-04	3.12E-04

Note: EA: Educational attainment

### MR analyses

MR analysis was performed by using the inverse-variance weighted (IVW) method to assess the effect of exposures (risk factors) on the outcome (disease). Then, this primary analysis was complemented with the weighted median and MR‒Egger methods [32]. We performed the overall effects of EA, intelligence, and income on the risks of SARS-CoV-2 infection as well as hospitalized and critical COVID-19 by MR. The intercept from the MR‒Egger regression was utilized to evaluate the average horizontal pleiotropy [33]. The heterogeneity in the MR analysis was evaluated by Cochran’s Q test and I^2^ statistics (both *P* < 0.05 and I^2^ > 0.25) [34]. The significant associations between EA, intelligence, income, and COVID-19 were determined by IVW-based FDR < 0.05. Single-nucleotide polymorphisms (SNPs) with genome-wide significance (*P* < 5 × 10^–8^) in the exposure dataset were selected as instrumental variables (IVs) and further pruned using a clumping r^2^ cutoff of 0.01 within a 10 Mb window. For each MR analysis, we removed SNPs not present in the outcome dataset and palindromic SNPs with intermediate allele frequencies.

### MVMR analysis

We analyzed the direct effects of EA, intelligence, and income on COVID-19 by MVMR. When a difference between the causal estimates of the MR (overall effects) and MVMR analysis (direct or independent causal effects) is found, it implies that the causal effect, at least in part, acts via potential mediators.

We conducted all the MR analyses in R (version 4.0.5) [35]. An FDR value below 0.05 was considered statistically significant in all analyses.

## Results

### Genetic correlation analysis

The genetic correlation analysis showed negative correlations of EA (r_g_: -0.28‒-0.36), intelligence (r_g_: -0.22‒-0.31), and income (r_g_: -0.19‒-0.26) with all three COVID-19 outcomes studied (Table 2).

### MR analysis

**Fig. 1:**
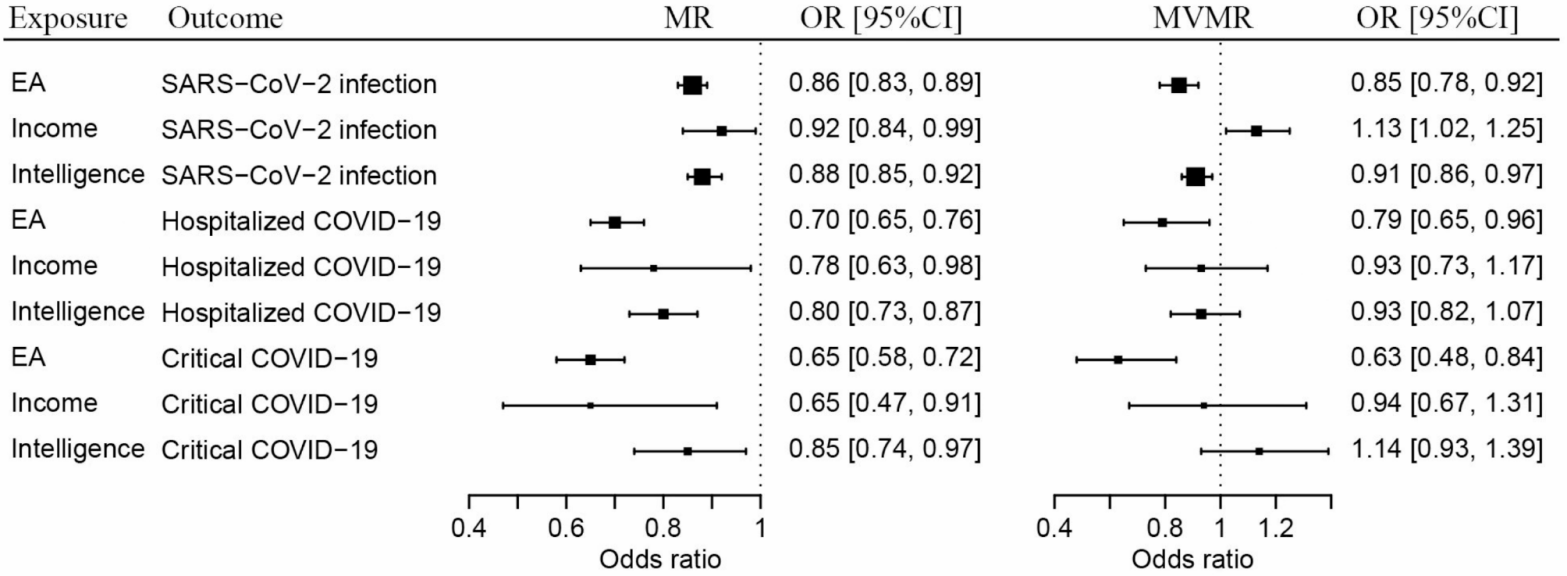
Forest plots showing the effect size of causal effects of educational attainment (EA), intelligence, and income on COVID-19 outcomes by MR and MVMR analyses

MR analysis demonstrated that higher EA conferred protective effects against SARS-CoV-2 infection (OR: 0.86, 95% confidence interval (CI): 0.83‒0.89, *P* = 3.67 × 10^− 20^), hospitalization for COVID-19 (0.70, 0.65‒0.76, *P* = 1.07 × 10^− 19^), and critical COVID-19 (0.65, 0.58‒0.72, *P* = 1.46 × 10^− 15^) (Table 3; Fig. 1).

**Table 3:** Causal effects of educational attainment, intelligence, and income on COVID-19 outcomes in univariable MR analyses

Exposure	Outcome	Method	b (se)	OR [95%CI]	N_IV	P_IV	Q	Q_P	I^2^	Egger_ intercept	P_ pleiotropy	*P*
EA	SARS-CoV-2 infection	IVW	-0.155 (0.017)	0.86 [0.83–0.89]	670	5.00E-08	8.24E + 02	3.50E-05	0.189	NA	NA	3.67E-20
EA	SARS-CoV-2 infection	WM	-0.159 (0.023)	0.85 [0.82–0.89]	670	5.00E-08	NA	NA	NA	NA	NA	5.27E-12
EA	SARS-CoV-2 infection	MR‒Egger	-0.113 (0.064)	0.89 [0.79–1.01]	670	5.00E-08	8.24E + 02	3.32E-05	0.188	-0.001	0.499	0.078
EA	hospitalized COVID-19	IVW	-0.350 (0.038)	0.70 [0.65–0.76]	667	5.00E-08	9.17E + 02	3.04E-10	0.274	NA	NA	1.07E-19
EA	hospitalized COVID-19	WM	-0.324 (0.052)	0.72 [0.65–0.80]	667	5.00E-08	NA	NA	NA	NA	NA	4.00E-10
EA	hospitalized COVID-19	MR‒Egger	-0.288 (0.146)	0.75 [0.56-1.00]	667	5.00E-08	9.17E + 02	2.67E-10	0.274	-0.001	0.661	0.05
EA	critical COVID-19	IVW	-0.434 (0.054)	0.65 [0.58–0.72]	672	5.00E-08	8.25E + 02	4.18E-05	0.187	NA	NA	1.46E-15
EA	critical COVID-19	WM	-0.474 (0.076)	0.62 [0.54–0.72]	672	5.00E-08	NA	NA	NA	NA	NA	4.06E-10
EA	critical COVID-19	MR‒Egger	-0.452 (0.209)	0.64 [0.42–0.96]	672	5.00E-08	8.25E + 02	3.74E-05	0.187	0	0.932	0.031
Intelligence	SARS-CoV-2 infection	IVW	-0.123 (0.022)	0.88 [0.85–0.92]	211	5.00E-08	3.20E + 02	1.57E-06	0.343	NA	NA	3.20E-08
Intelligence	SARS-CoV-2 infection	WM	-0.139 (0.027)	0.87 [0.83–0.92]	211	5.00E-08	NA	NA	NA	NA	NA	3.65E-07
Intelligence	SARS-CoV-2 infection	MR‒Egger	-0.201 (0.101)	0.82 [0.67-1.00]	211	5.00E-08	3.19E + 02	1.50E-06	0.341	0.002	0.429	0.048
Intelligence	hospitalized COVID-19	IVW	-0.226 (0.044)	0.80 [0.73–0.87]	211	5.00E-08	2.76E + 02	1.44E-03	0.24	NA	NA	3.75E-07
Intelligence	hospitalized COVID-19	WM	-0.228 (0.061)	0.80 [0.71–0.90]	211	5.00E-08	NA	NA	NA	NA	NA	1.83E-04
Intelligence	hospitalized COVID-19	MR‒Egger	-0.194 (0.202)	0.82 [0.55–1.22]	211	5.00E-08	2.76E + 02	1.24E-03	0.24	-0.001	0.873	0.338
Intelligence	critical COVID-19	IVW	-0.167 (0.068)	0.85 [0.74–0.97]	211	5.00E-08	2.92E + 02	1.45E-04	0.282	NA	NA	0.015
Intelligence	critical COVID-19	WM	-0.151 (0.090)	0.86 [0.72–1.03]	211	5.00E-08	NA	NA	NA	NA	NA	0.093
Intelligence	critical COVID-19	MR‒Egger	-0.163 (0.311)	0.85 [0.46–1.56]	211	5.00E-08	2.92E + 02	1.21E-04	0.282	0	0.991	0.6
income	SARS-CoV-2 infection	IVW	-0.088 (0.042)	0.92 [0.84–0.99]	89	5.00E-08	1.59E + 02	6.03E-06	0.445	NA	NA	0.035
income	SARS-CoV-2 infection	WM	-0.098 (0.049)	0.91 [0.82-1.00]	89	5.00E-08	NA	NA	NA	NA	NA	0.046
income	SARS-CoV-2 infection	MR‒Egger	0.229 (0.185)	1.26 [0.87–1.81]	89	5.00E-08	1.53E + 02	1.57E-05	0.425	-0.006	0.083	0.219
income	hospitalized COVID-19	IVW	-0.243 (0.115)	0.78 [0.63–0.98]	89	5.00E-08	2.63E + 02	2.54E-19	0.665	NA	NA	0.034
income	hospitalized COVID-19	WM	-0.326 (0.106)	0.72 [0.59–0.89]	89	5.00E-08	NA	NA	NA	NA	NA	2.20E-03
income	hospitalized COVID-19	MR‒Egger	0.324 (0.504)	1.38 [0.52–3.71]	89	5.00E-08	2.59E + 02	5.57E-19	0.66	-0.011	0.251	0.522
income	critical COVID-19	IVW	-0.423 (0.166)	0.65 [0.47–0.91]	88	5.00E-08	2.36E + 02	1.25E-15	0.631	NA	NA	0.011
income	critical COVID-19	WM	-0.676 (0.160)	0.51 [0.37–0.70]	88	5.00E-08	NA	NA	NA	NA	NA	2.28E-05
income	critical COVID-19	MR‒Egger	0.177 (0.748)	1.19 [0.28–5.17]	88	5.00E-08	2.34E + 02	1.36E-15	0.628	-0.011	0.413	0.814

Higher intelligence was associated with decreased risks for SARS-CoV-2 infection (0.88, 0.85‒0.92, *P* = 3.20 × 10^− 8^), hospitalized COVID-19 (0.80, 0.73‒0.87, *P* = 3.75 × 10^− 7^), and critical COVID-19 (0.85, 0.74‒0.97, *P* = 0.015) (Table 3; Fig. 1).

Higher income was associated with decreased risks of SARS-CoV-2 infection (0.92, 0.84‒0.99, *P* = 0.035), hospitalized COVID-19 (0.78, 0.63‒0.98, *P* = 0.034) and critical COVID-19 (0.65, 0.47‒0.91, *P* = 0.011) (Table 3; Fig. 1).

### Sensitivity analysis

Three methods of MR analyses were performed to test the sensitivity of the effects of the exposure on the outcomes. The causal effects of EA, intelligence, and income on the outcomes of COVID-19 were similar across the three methods. The effect sizes were between 0.51 and 1.38 (Table 3).

### MVMR analysis

In the MVMR analysis, associations of higher EA with decreased risks for SARS-CoV-2 infection (OR = 0.85, 95% CI: 0.78‒0.92, *P* = 1.44 × 10^− 4^), hospitalized COVID-19 (0.79, 0.65‒0.96, *P* = 0.020), and critical COVID-19 (0.63, 0.48‒0.84, *P* = 1.52 × 10^− 3^) remained significant after controlling for the effects of intelligence and income (Table 4; Fig. 1).

**Table 4:** Causal effects of educational attainment, intelligence, and income on COVID-19 outcomes in multivariable MR analyses

Exposure	Outcome	N_IV	b (se)	OR (95%CI)	*P*	FDR
EA	SARS-CoV-2 infection	603	-0.17 (0.04)	0.85 (0.78–0.92)	1.44E-04	0.0013
Income	SARS-CoV-2 infection	49	0.12 (0.05)	1.13 (1.02–1.25)	0.022	0.039
Intelligence	SARS-CoV-2 infection	143	-0.09 (0.03)	0.91 (0.86–0.97)	2.33E-03	0.007
EA	Hospitalized COVID-19	601	-0.23 (0.10)	0.79 (0.65–0.96)	0.020	0.039
Income	Hospitalized COVID-19	49	-0.08 (0.12)	0.93 (0.73–1.17)	0.516	0.580
Intelligence	Hospitalized COVID-19	143	-0.07 (0.07)	0.93 (0.82–1.07)	0.333	0.429
EA	Critical COVID-19	605	-0.46 (0.14)	0.63 (0.48–0.84)	1.52E-03	0.00684
Income	Critical COVID-19	49	-0.07 (0.17)	0.94 (0.67–1.31)	0.700	0.700
Intelligence	Critical COVID-19	143	0.13 (0.10)	1.14 (0.93–1.39)	0.200	0.300

Note: EA: Educational attainment

Higher intelligence remained associated with a decreased risk for SARS-CoV-2 infection (0.91, 0.86‒0.97, *P* = 2.33 × 10^− 3^), while its association with hospitalized COVID-19 (0.93, 0.82‒1.07, *P* = 0.333) and critical COVID-19 (1.14, 0.93‒1.39, *P* = 0.200) lost its significance after controlling for the effects of EA and income (Table 4; Fig. 1).

Interestingly, higher income was associated with an increased risk for SARS-CoV-2 infection (1.13, 1.02‒1.25, *P* = 0.022), while exerting no causal effects on hospitalized COVID-19 (0.93, 0.73‒1.17, *P* = 0.516) and critical COVID-19 (0.94, 0.67‒1.31, *P* = 0.700), when the other two factors were taken into account (Table 4; Fig. 1). The reversal of the causal relationship between income and SARS-CoV-2 infection and the loss of income’s effect on hospitalized COVID-19 could be partially explained by the strong protective effects of EA and intelligence on SARS-CoV-2 infection, which may compensate for the detrimental effects of high income.

## Discussion

As the first step, we examined the genetic correlations between the three psychosocial factors and COVID-19 outcomes and found that each of the three psychosocial factors was negatively correlated with each outcome of COVID-19 (Table 2). Then, we evaluated the overall causal effects of EA, intelligence, and income on COVID-19 outcomes and found that they could protect against SARS-CoV-2 infection, hospitalized COVID-19, and critical COVID-19 (Table 3; Fig. 1). Finally, we tested their independent effects on COVID-19 outcomes and found that only EA could protect against all COVID-19 outcomes independently. Intelligence protected against SARS-CoV-2 infection but not against severe forms of COVID-19, while higher income was found to increase the risks of SARS-CoV-2 infection independently of two other socioeconomic factors (Table 4; Fig. 1).

To date, three MR studies have investigated the relationships between psychosocial factors and COVID-19. The first MR study reported that higher EA confers a lower risk of severe COVID-19 [24]. The second study revealed that both EA and intelligence exerted causal effects on hospitalized and critical COVID-19 outcomes in either MR or MVMR analysis but did not evaluate the effects of income [26]. The third study, based on MVMR, found that EA exerts protective effects on SARS-CoV-2 infection as well as on hospitalized and critical COVID-19 patients but did not report the effects of intelligence and income on any COVID-19 outcomes [25]. Our study differed from previous studies described above in employing the most recent GWAS datasets and in testing the overall and independent effects of EA, intelligence, and income on each outcome of COVID-19.

When the independent effects of each psychosocial factor were evaluated, some surprising discoveries were made. In particular, we found that genetic predisposition to higher intelligence might reduce the risk of SARS-CoV-2 infection but not severe COVID-19. People with high intelligence may be better able to understand defensive measures, which may translate into a more efficient assessment of personal risks and, as a result, lessening one’s chances of being infected. On the other hand, recent evidence suggests that COVID-19 may exert a detrimental effect on memory and intelligence [36].

We confirmed previous findings suggesting that the genetic underpinning of high EA contributed to reducing exposure to COVID-19 and the propensity to develop its severe forms. People with higher education may exercise better long-term strategies for the management of their health by maintaining a risk-reducing lifestyle [37, 38]. Even if infected, a better baseline physique may prevent significant deterioration of health. A previous observational study found that higher EA was associated with a lower propensity to smoke, to be inactive or obese, or to have hypertension, hypercholesterolemia, hyperglycemia, and high BMI [39, 40]. It was found that comorbidity with other somatic diseases, such as cancer, cardiovascular diseases, cerebrovascular diseases, or diabetes, was associated with increased severity and mortality of COVID-19 [4, 9, 41]. In accordance with these observations, we found that intelligence itself cannot protect against the severity of COVID-19, but higher EA may decrease the severity of SARS-CoV-2 disease outcomes independent of intelligence.

Remarkably, the protective effects of income on SARS-CoV-2 infection were reversed in the MVMR analysis. Higher income was associated with an increased risk for SARS-CoV-2 infection, and its protective effects on hospitalization disappeared. Our study suggested that the protective effects of higher income on COVID-19 were mainly mediated by education. A combination of high income with low EA might be counterproductive for efforts against infections for a couple of reasons. First, the high possibility of infection in high-income populations might be due to their higher mobility in the early stage of the pandemic, when they retained the ability to travel between different countries or areas, thus increasing the risks of contracting the virus. Second, high-income populations might have greater access to molecular diagnostics, which increased COVID-19 positivity rates in these groups [42].

During the COVID-19 pandemic, higher rates of infection and death from COVID-19 in low-income or middle-income populations have been reported repeatedly [43]. A study of COVID-19 inpatients in UK hospitals showed that larger social deprivation scores were associated with a higher risk of death [44]. In Sweden, being a man, having a lower personal income, having lower education, and being single increased the risk of death from COVID-19 [45]. The higher rates of hospitalization, severity, and mortality of COVID-19 in the populations of low socioeconomic status may be due to poor housing, physically exhausting jobs, worse hygiene, low food quality, more substance misuse, less access to healthcare systems, and delayed contact with the health care provider, along with difficulties in affording the expense of medical care [19, 46]. Accordingly, in developed countries, the mortality and morbidity of SARS-CoV-2 were significantly higher in ethnic minorities than in Caucasians [47]. Most ethnic minorities were of lower socioeconomic status, and their education levels and income levels were not high.

This study was not free of limitations. First, the current MR analysis employed the summary statistics of GWAS meta-analyses conducted among Europeans, indicating that the causality inferred from these datasets might apply to Europeans only. Second, we only analyzed EA, intelligence, income, and COVID-19, while other sociodemographic factors and clinical parameters were not examined. Third, we realized that the outcomes of SARS-CoV-2 infection depended on the individuals’ overall situations, which included sociodemographic characteristics, comorbidities, immune status, and anthropometrics rather than being solely due to genetics.

To summarize, our study affirmed that EA had a beneficial impact on reducing vulnerability to SARS-CoV-2 and lessening the severity of COVID-19. Importantly, these effects were not influenced by intelligence and income. Nevertheless, our study did not find evidence supporting the individual effects of intelligence or income on the severity of COVID-19.

## Electronic supplementary material

Below is the link to the electronic supplementary material.

Supplementary Material 1

## Data Availability

No datasets were generated or analysed during the current study.
