# Supplementary material for: Causal effects of education, intelligence, and income on COVID-19: evidence from a Mendelian randomization study
Source: Hum Genomics. 2025 Feb 25;19:18. doi: 10.1186/s40246-025-00731-y (PMC11863516; doi:10.1186/s40246-025-00731-y)
Supplement: Supplementary file 1 — Supplementary Material 1 [file 40246_2025_731_MOESM1_ESM.docx]

**Supplement**

Causal effects of education, intelligence, and income on COVID-19: evidence from a Mendelian randomization study

by Yuqing Song, Ancha Baranova, Hongbao Cao, Weihua Yue, Fuquan Zhang

**Table S1 STROBE-MR checklist of recommended items to address in reports of Mendelian randomization studies**^1^ ^2^

| **Item No.** | **Section** | **Checklist item** | **Page No.** | **Relevant text from manuscript** |
| --- | --- | --- | --- | --- |
| 1 | **TITLE and ABSTRACT** | Indicate Mendelian randomization (MR) as the study’s design in the title and/or the abstract if that is a main purpose of the study. | 1-2 | Title: Causal effect of education, intelligence, and income on COVID-19: evidence from Mendelian randomization study |
|  | **INTRODUCTION** |  |  |  |
| 2 | **Background** | Explain the scientific background and rationale for the reported study. What is the exposure? Is a potential causal relationship between exposure and outcome plausible? Justify why MR is a helpful method to address the study question. | 3-4 | Observational studied showed psychosocial factors influence the outcomes of COVID-19.  Exposure: education attainment, intelligence and income.  Outcome: SARS-CoV-2 infection, hospitalized COVID-19, critical COVID-19 |
| 3 | **Objectives** | State specific objectives clearly, including prespecified causal hypotheses (if any). State that MR is a method that, under specific assumptions, intends to estimate causal effects. | 4 | The hypotheses are education attainment, intelligence and income can protect from outcome of COVID-19. |
|  | **METHODS** |  | 5-6 |  |
| 4 | **Study design and data sources** | Present key elements of the study design early in the article. Consider including a table listing sources of data for all phases of the study. For each data source contributing to the analysis, describe the following: | 5 | Using the publicly available GWAS data from European population.  See in Table 1. |
|  | a) | Setting: Describe the study design and the underlying population, if possible. Describe the setting, locations, and relevant dates, including periods of recruitment, exposure, follow-up, and data collection, when available. | 5 | Using the publicly available GWAS data from European population.  See in Table 1. |
|  | b) | Participants: Give the eligibility criteria, and the sources and methods of selection of participants. Report the sample size, and whether any power or sample size calculations were carried out prior to the main analysis. | 5 | Including SARS-CoV-2 infection (122,616 cases and 2,475,240 controls), hospitalized COVID-19 (32,519 cases and 2,062,805 controls), and critical COVID-19 (13,769 cases and 1,072,442 controls). The GWAS dataset for EA, intelligence, and household income included 765283, 269867, and 392422 participants, respectively. |
|  | c) | Describe measurement, quality control and selection of genetic variants | 5 |  |
|  | d) | For each exposure, outcome, and other relevant variables, describe methods of assessment and diagnostic criteria for diseases. | 5 |  |
|  | e) | Provide details of ethics committee approval and participant informed consent, if relevant. | NA |  |
| 5 | **Assumptions** | Explicitly state the three core IV assumptions for the main analysis (relevance, independence and exclusion restriction) as well assumptions for any additional or sensitivity analysis. | NA |  |
| 6 | **Statistical methods: main analysis** | Describe statistical methods and statistics used | 5-6 | Genetic correlation, IVW, weighted median and MR‒Egger methods, multivariable Mendelian randomization (MVMR) analyses. |
|  | a) | Describe how quantitative variables were handled in the analyses (i.e., scale, units, model). | 5-6 | . |
|  | b) | Describe how genetic variants were handled in the analyses and, if applicable, how their weights were selected. | 5-6 | A set of SNPs was filtered down to 1.1 million variants, a subset of 1000 Genomes and HapMap3, with MAF above 0.05. Significant genetic correlations were determined after applying the correction for the false discovery rate (FDR < 0.05) |
|  | c) | Describe the MR estimator (e.g. two-stage least squares, Wald ratio) and related statistics. Detail the included covariates and, in case of two-sample MR, whether the same covariate set was used for adjustment in the two samples. | 5-6 | Genetic correlation, IVW, weighted median and MR‒Egger methods, multivariable Mendelian randomization (MVMR) analyses. |
|  | d) | Explain how missing data were addressed. | 6 | we removed SNPs not present in the outcome dataset and palindromic SNPs with intermediate allele frequencies. |
|  | e) | If applicable, indicate how multiple testing was addressed. | 5-6 | multivariable Mendelian randomization (MVMR) analyze |
| 7 | **Assessment of assumptions** | Describe any methods or prior knowledge used to assess the assumptions or justify their validity. | NA |  |
| 8 | **Sensitivity analyses and additional analyses** | Describe any sensitivity analyses or additional analyses performed (e.g. comparison of effect estimates from different approaches, independent replication, bias analytic techniques, validation of instruments, simulations). | 5-6 | Two-sample Mendelian randomization (MR) analysis was performed by using the inverse-variance weighted (IVW) method to assess the effect of exposure (risk factors) on the outcome (disease), complemented with the weighted median and MR‒Egger methods. |
| 9 | **Software and preregistration** |  | 6 |  |
|  | a) | Name statistical software and package(s), including version and settings used. | 6 | We conducted all the MR analyses in R (version 4.0.5) |
|  | b) | State whether the study protocol and details were preregistered (as well as when and where). | NA |  |
|  | **RESULTS** |  | 6-7 |  |
| 10 | **Descriptive data** |  |  |  |
|  | a) | Report the numbers of individuals at each stage of included studies and reasons for exclusion. Consider use of a flow diagram. | NA |  |
|  | b) | Report summary statistics for phenotypic exposure(s), outcome(s), and other relevant variables (e.g. means, SDs, proportions). | NA |  |
|  | c) | If the data sources include meta-analyses of previous studies, provide the assessments of heterogeneity across these studies. | NA |  |
|  | d) | For two-sample MR:  i. Provide justification of the similarity of the genetic variant-exposure associations between the exposure and outcome samples.  ii. Provide information on the number of individuals who overlap between the exposure and outcome studies. | NA |  |
| 11 | **Main results** |  |  |  |
|  | a) | Report the associations between genetic variant and exposure, and between genetic variant and outcome, preferably on an interpretable scale. | 6-7 | The genetics of EA, intelligence, and income were highly positively correlated with each other .Three of which negative correlated with the three COVID-19 outcomes. |
|  | b) | Report MR estimates of the relationship between exposure and outcome, and the measures of uncertainty from the MR analysis, on an interpretable scale, such as odds ratio or relative risk per SD difference. | 6-7 | The MR analysis indicated that EA, intelligence, and income exerted strong bidirectional causal effects on one another.  Genetic liability to EA, intelligence, and income exerted overall protective effects on SARS-CoV-2 infection and hospitalized COVID-19; EA was also associated with a decreased risk of critical COVID-19.  In the MVMR analysis, higher EA conferred independent protective effects on COVID-19 outcomes. Intelligence was associated with a decreased risk for SARS-CoV-2 infection, while high income was associated with an increased risk of SARS-CoV-2 infection. |
|  | c) | If relevant, consider translating estimates of relative risk into absolute risk for a meaningful time period. | NA |  |
|  | d) | Consider plots to visualize results (e.g. forest plot, scatterplot of associations between genetic variants and outcome versus between genetic variants and exposure). | 24 | See figure 1. |
| 12 | **Assessment of assumptions** |  |  |  |
|  | a) | Report the assessment of the validity of the assumptions. | 20 | See table 3. |
|  | b) | Report any additional statistics (e.g., assessments of heterogeneity across genetic variants, such as *I^2^*, Q statistic or E-value). | 20 | Table 3 listed the results. |
| 13 | **Sensitivity analyses and additional analyses** |  |  |  |
|  | a) | Report any sensitivity analyses to assess the robustness of the main results to violations of the assumptions. | 7,20 | Three MR methods (IVW, weighted median and MR‒Egger methods) were used to test the causal relationship between exposures and outcomes. |
|  | b) | Report results from other sensitivity analyses or additional analyses. | 7,20 | The results of three methods were written and list in table 3. |
|  | c) | Report any assessment of direction of causal relationship (e.g., bidirectional MR). | 6-7 | The MR analysis indicated that EA, intelligence, and income exerted strong bidirectional causal effects on one another. |
|  | d) | When relevant, report and compare with estimates from non-MR analyses. | NA |  |
|  | e) | Consider additional plots to visualize results (e.g., leave-one-out analyses). | 24 | Forest plot was given in the manuscript. |
|  | **DISCUSSION** |  | 8-10 |  |
| 14 | **Key results** | Summarize key results with reference to study objectives. | 8 | 1.Genetic correlations between the three psychosocial factors and found that they were highly positively correlated with each other. The three psychosocial factors were negatively correlated with each outcome of COVID-19 in genetics .  2.The bidirectional effects showed EA, intelligence, and income having causal effects between them.  3. EA, intelligence, and income had overall causal effects on COVID-19 outcomes and they could protect against SARS-CoV-2 infection and hospitalized COVID-19, but only EA could protect them from critical COVID-19.  4. EA could protected against all COVID-19 outcomes independently. Intelligence protected against SARS-CoV-2 infection but not against severe forms of COVID-19, while higher income was found to increase the risks of SARS-CoV-2 infection independently of two other socioeconomic factors. |
| 15 | **Limitations** | Discuss limitations of the study, taking into account the validity of the IV assumptions, other sources of potential bias, and imprecision. Discuss both direction and magnitude of any potential bias and any efforts to address them | 10 | 1. The current MR analysis employed the summary statistics of GWAS meta-analyses conducted among Europeans, indicating that the causality inferred from these datasets might apply to Europeans only.  2. We only analyzed EA, intelligence, income, and COVID-19; other sociodemographic factors and clinical parameters were not examined.  3. We realized that the outcomes of SARS-CoV-2 infection depended on the individuals’ overall situations, which included sociodemographic characteristics, comorbidities, immune status, and anthropometrics rather than solely genetics. |
| 16 | **Interpretation** |  |  |  |
|  | a) | Meaning: Give a cautious overall interpretation of results in the context of their limitations and in comparison with other studies. | 10 | First, the current MR analysis employed the summary statistics of GWAS meta-analyses conducted among Europeans, indicating that the causality inferred from these datasets might apply to Europeans only. Second, we only analyzed EA, intelligence, income, and COVID-19; other sociodemographic factors and clinical parameters were not examined. Third, we realized that the outcomes of SARS-CoV-2 infection depended on the individuals’ overall situations, which included sociodemographic characteristics, comorbidities, immune status, and anthropometrics rather than solely genetics. |
|  | b) | Mechanism: Discuss underlying biological mechanisms that could drive a potential causal relationship between the investigated exposure and the outcome, and whether the gene‒environment equivalence assumption is reasonable. Use causal language carefully, clarifying that IV estimates may provide causal effects only under certain assumptions. | 8-10 |  |
|  | c) | Clinical relevance: Discuss whether the results have clinical or public policy relevance, and to what extent they inform effect sizes of possible interventions. | 10 | During virus pandemic period, population with lower socioeconomic status (lower education, income et al) should be concerned particularly. |
| 17 | **Generalizability** | Discuss the generalizability of the study results (a) to other populations, (b) across other exposure periods/timings, and (c) across other levels of exposure. | 10 | The current MR analysis employed the summary statistics of GWAS meta-analyses conducted among Europeans, indicating that the causality inferred from these datasets might apply to Europeans only. |
|  | **OTHER INFORMATION** |  |  |  |
| 18 | **Funding** | Describe sources of funding and the role of funders in the present study and, if applicable, sources of funding for the databases and original study or studies on which the present study is based. | 11 | This research received no external funding. |
| 19 | **Data and data sharing** | Provide the data used to perform all analyses or report where and how the data can be accessed, and reference these sources in the article. Provide the statistical code needed to reproduce the results in the article, or report whether the code is publicly accessible and if so, where. | 5,11,18 | See table 1 |
| 20 | **Conflicts of Interest.** | All authors should declare all potential conflicts of interest. | 11 | We declare no potential conflict of interest. |

This checklist is copyrighted by the Equator Network under the Creative Commons Attribution 3.0 Unported (CC BY 3.0) license.

Supplementary references:

1. Skrivankova VW, Richmond RC, Woolf BAR, Yarmolinsky J, Davies NM, Swanson SA, et al. Strengthening the Reporting of Observational Studies in Epidemiology using Mendelian Randomization (STROBE-MR) Statement. JAMA. 2021;under review.

2. Skrivankova VW, Richmond RC, Woolf BAR, Davies NM, Swanson SA, VanderWeele TJ, et al. Strengthening the Reporting of Observational Studies in Epidemiology using Mendelian Randomization (STROBE-MR): Explanation and Elaboration. BMJ. 2021;375:n2233.
